# Microleakage and Torque Loss at the Implant–Abutment Interface in Original Versus Non-Original Abutments: An In Vitro Study

**DOI:** 10.3390/ma19091884

**Published:** 2026-05-02

**Authors:** Ferran Sánchez-Benito, Enrique Castells-Mira, María Cosin-Villanueva, Francisco Gil-Loscos, Andrés López-Roldán

**Affiliations:** Department of Stomatology, Faculty of Medicine and Dentistry, University of Valencia, 46010 Valencia, Spain; sanbefe@alumni.uv.es (F.S.-B.); enriquecastellsmira@gmail.com (E.C.-M.); maria.cosin@uv.es (M.C.-V.); francisco.j.gil@uv.es (F.G.-L.)

**Keywords:** microleakage, implant–abutment interface, prosthetic abutments, hydraulic conductance, torque loss, dental implants

## Abstract

**Highlights:**

- Non-original abutments showed significantly greater microleakage than original components.
- Reduced tightening torque (5 N·cm) significantly increased microleakage values.
- Microleakage showed a significant correlation with abutment screw torque loss.
- Original abutments provided improved sealing at the implant–abutment interface.
- Manufacturer-recommended torque (35 N·cm) minimized microleakage.
- Hydraulic conductance perfusion enables quantitative microleakage assessment.

**Abstract:**

Microleakage at the implant–abutment interface represents a potential pathway for bacterial penetration and may contribute to peri-implant inflammation, marginal bone loss, and mechanical complications such as screw loosening. The increasing clinical use of compatible prosthetic abutments as cost-effective alternatives to original components has raised concerns regarding their fit, sealing capacity, and mechanical stability at this interface. The aim of this in vitro study was to evaluate differences in sealing capacity and torque loss between original and non-original abutments in a mixed internal connection implant system and to investigate the applicability of a novel quantitative approach for assessing microleakage based on a hydraulic conductance perfusion system. Nine abutments, including four multi-unit and five screw-retained cementable abutments, were connected to Straumann Bone Level implants at two tightening torques (5 N·cm and 35 N·cm). Microleakage was quantified by measuring fluid transport across the implant–abutment interface using the perfusion system, and removal torque values were recorded after testing. Non-original abutments exhibited significantly greater microleakage than original abutments at both torque levels. Microleakage increased significantly when the installation torque was reduced to 5 N·cm. At the manufacturer-recommended torque, screw-retained cementable abutments demonstrated higher microleakage than multi-unit abutments. Non-original abutments also showed significantly greater torque loss. These findings suggest that original abutments provide improved sealing capacity and mechanical stability at the implant–abutment interface, while the hydraulic conductance perfusion system represents a promising quantitative tool for investigating microleakage.

## 1. Introduction

The implant–abutment prosthetic connection relies on mechanical interlocking between two distinct structures, resulting in the presence of a certain degree of discrepancy and mechanical microtolerance. In addition, the interface and the implant neck are the primary areas of stress concentration during functional loading, constituting a critical zone within the system from both mechanical and biologic perspectives [1,2]. Physically, a microgap—generally ranging from 6 to 10 µm—exists at this junction, allowing for fluid penetration, bacterial colonization and microbial proliferation [3,4].

A precise fit between components is essential for successful outcomes [5], particularly in submerged implants, as the establishment of a microgap in proximity to the alveolar bone is a recognized risk factor for early bone loss and peri-implantitis [6,7,8,9,10,11]. Misfit may compromise system passivity and may contribute to abutment screw loosening, deformation or fracture of prosthetic components, crestal bone loss, and implant failure [12,13,14]. Additionally, microleakage through the microgap may contribute to progressive loosening of the fixation screw [15].

The microgap between components creates a bacterial reservoir, inducing soft-tissue inflammation adjacent to the implant–abutment interface [16].

Despite preventive efforts, bacterial colonization of the microgap is a well-documented phenomenon [3,4,17,18,19]. However, the extent of bacterial colonization depends on several factors, including the accuracy of fit, the torque applied during connection, the frequency of loosening and retightening, and the functional loading of the implant.

Because the implant–abutment interface is located near the alveolar crest, bacterial presence within the microgap has been considered a factor in the establishment of biologic width and, consequently, early marginal bone loss [6,7]. Due to the inflammatory stimulus generated by the microgap, soft tissues attach apically to the interface [20], requiring a minimum soft-tissue thickness of approximately 3 mm to achieve peri-implant tissue stability.

A further determinant of marginal bone loss is micromovement between the abutment and the implant under functional loading. This movement creates volumetric changes within the implant system, transporting microorganisms that were initially immobile from the exterior to the interior and vice versa [21]. This phenomenon, known as the pumping effect, acts as a chronic driver of inflammation and marginal bone loss [8,9]. Furthermore, abutment–implant micromovements during function have also been associated with implant failure [22,23], as well as component fractures in 5–20% of cases [24,25].

Currently, no two-piece implant system provides a complete seal at the connection, and therefore bacterial microleakage is a universal finding [26]. Moreover, the selection of non-original abutments by clinicians and dental technicians may introduce variances in connection surface design, shape, dimensions, and materials; these components have been shown to exhibit higher microleakage values [27].

The use of non-original abutments may also increase micromovements at the implant–abutment interface, increasing stress at the marginal bone level and enhancing the pumping effect [21]. Misfit between original and non-original components may also occur in other parts of the system, such as abutment fixation screws [28], resulting in greater rotational misfit as well as significant differences in groove and surface combinations [29]. This has mechanical and biologic implications, as non-original components have been reported to exhibit lower mechanical resistance and increased microleakage, potentially compromising long-term implant survival.

Various methodologies exist to measure the size of the implant–abutment microgap and associated microleakage in vitro, microscopy or radiographic techniques such as micro-computed tomography, as well as dye microleakage assessed by spectrophotometry or bacterial inoculation [19,30]. However, the latter methods may be influenced by trapped air and lead to inaccurate conclusions [31]. Consequently, the application and adaptation of in vitro methods originally developed to evaluate dentin hydraulic conductance [32] has been proposed as a promising alternative. This approach offers sensitive, quantitative, and reproducible data in a fluid-saturated environment similar to the oral cavity [15].

Despite extensive research, there is limited quantitative evidence comparing original and non-original abutments under controlled conditions. Moreover, current methods for assessing microleakage have limitations in sensitivity and reproducibility, highlighting the need for a reliable quantitative approach.

The purpose of this in vitro study was to evaluate differences in sealing capacity between original and non-original abutments in a mixed internal connection implant system. In addition, a quantitative approach based on hydraulic conductance perfusion was applied to assess microleakage under controlled conditions. Furthermore, the relationship between microleakage and torque loss was investigated to provide insight into the mechanical implications of interface leakage.

## 2. Materials and Methods

### 2.1. Study Design

This in vitro study was conducted at the Department of Stomatology, Faculty of Medicine and Dentistry, University of Valencia (Valencia, Spain). The authors followed the CRIS guidelines (Checklist for Reporting In Vitro Studies). The implant selected for the study was the Straumann Bone Level^®^ implant (Institut Straumann AG, Basel, Switzerland), featuring a 15-degree conical–cylindrical internal connection (CrossFit^®^) with a friction fit established by 4 internal grooves. Two implants (Ø4.1 mm; lots CK278 and CK358) were utilized for the experimental setup. Two categories of prosthetic abutments were evaluated: original components and non-original (compatible) components. Table 1 details the specifications of the nine abutments used, including manufacturing method, provenance, and manufacturer. All multi-unit abutments had a diameter of Ø4.6 mm, with gingival heights ranging from 2.5 to 3.5 mm.

### 2.2. Specimen Fabrication

A solid methacrylate cylinder (63 mm length, 24 mm width, 25 mm diameter) was fabricated using a parallel lathe (WM280V-F; Weiss Machine & Tools) to serve as the fixation base. Implant beds (Ø4.1 mm) were prepared on the superior surface of the specimen, spaced 10 mm apart, using a parallel milling machine (JMD-2; JET).

To facilitate fluid dynamics within the system, a Ø2 mm high-speed steel (HSS) drill was used at 800 rpm to create a central channel through the specimen. Subsequently, the implant bed was prepared using a Ø3.45 mm HSS drill to a depth of 12 mm, followed by a Straumann Ø3.5 mm drill at 300 rpm and a Straumann Ø4.1 mm tap at 15 rpm. Finally, a Straumann implant dummy was manually inserted and removed five times at each site to ensure precise thread adaptation. This procedure was performed exclusively to standardize the implant bed. The dummy implants were not used for experimental measurements.

On the inferior surface, threaded ports were prepared using a Ø3.45 mm HSS drill to a depth of 4 mm to allow connection of the perfusion system. All internal surfaces were cleaned with compressed air prior to assembly.

### 2.3. Sample Preparation

To enable hydraulic conductance testing, the apical ends of the Straumann Bone Level^®^ implants were perforated along the long axis using a Ø0.95 mm HSS drill. As the fixation screw diameter is Ø 1.35 mm, the internal threads remained intact, preserving the mechanical integrity of the screw channel.

### 2.4. Implant and Abutment Insertion

**Figure 1:**
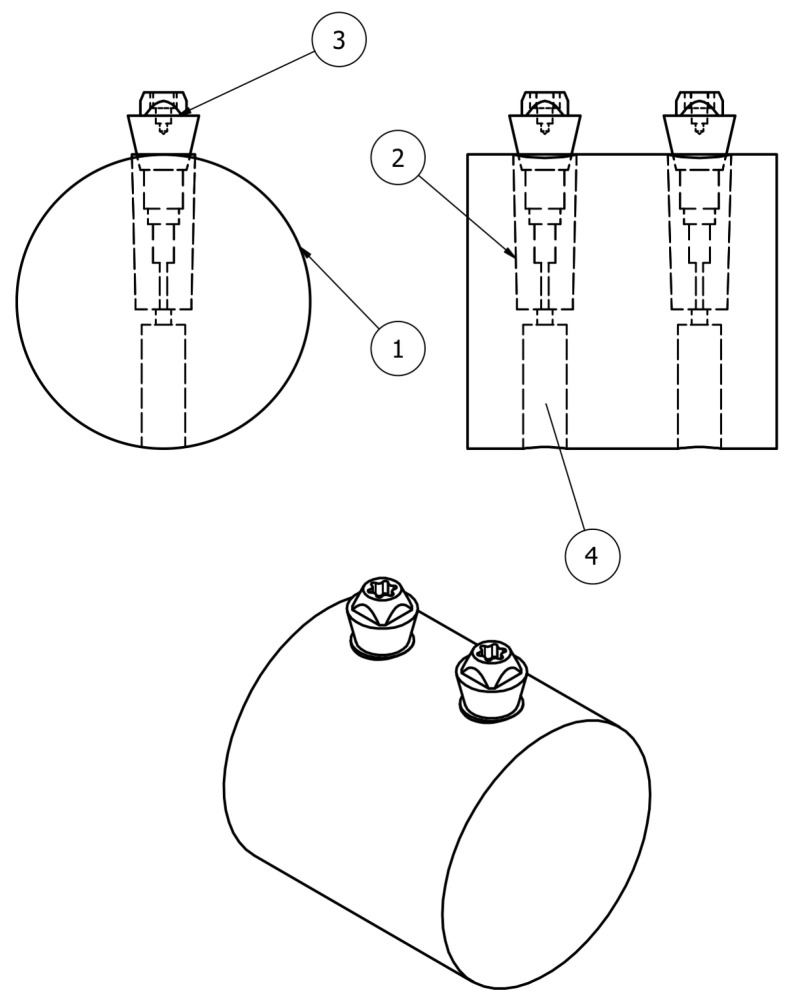
Schematic representation of two multi-unit abutments connected to implants inserted into the specimen. (1) Fixation base; (2) implant; (3) abutment; (4) water.

Implant insertion into the methacrylate base was performed following the manufacturer’s protocol using a ratchet wrench. The implant was connected to the Loxim™ insertion device, removed from the carrier, and positioned in the implant bed. It was advanced clockwise until final seating, ensuring correct orientation of the prosthetic interface according to the transfer markings. Insertion torque was controlled and did not exceed 35 N·cm, in accordance with the manufacturer’s recommendations. The Loxim™ device was removed after complete insertion. Prosthetic abutments were connected using the same torque wrenches, applying tightening torques of 5 N·cm and 35 N·cm according to the manufacturers’ instructions.” Prior to final seating, cyanoacrylate adhesive was applied to the middle threads of the implants, corresponding to the region indicated by label (2) in Figure 1, to hermetically seal the implant–bed interface, thereby preventing leakage external to the implant–abutment interface (IAI).

Prosthetic abutments were connected to the implants using two distinct torque values: the manufacturer-recommended torque (35 N·cm) and a sub-optimal torque (5 N·cm).

The reduced torque value (5 N·cm) was selected to simulate a condition of insufficient preload associated with manual tightening. This approach allows evaluation of the sealing capacity of the system under compromised mechanical conditions.

Tightening was performed using manufacturer-specific torque wrenches (Table 1), which were new and used according to manufacturer specifications, ensuring consistent torque application.

Two Straumann^®^ Bone Level implants and nine prosthetic abutments were evaluated, divided into 2 groups:

- Group 1: Multi-unit abutments (*n* = 4).
- Group 2: Screw-retained cementable abutments (*n* = 5).

Each abutment was tested on both implants in four separate trials: twice at a tightening torque of 5 N·cm and twice at 35 N·cm.

### 2.5. Hydraulic Conductance Perfusion System

The perfusion system was designed to quantify microleakage via fluid volume transport. It comprised a filtration fluid reservoir, a glass distributor, latex tubing, graduated glass micropipettes, and the specimen block.

- The fluid reservoir consisted of a separatory funnel which served as the hydrostatic pressure force. The height of the reservoir was adjustable to regulate pressure; the upper chamber contained deionized water, while the lower outlet connected to a latex tube (1 cm^2^ cross-section) leading to the distributor.
- The glass distributor featured 10 outlets; two were used to channel fluid to the specimen via latex and silicone tubing (diameter 1 cm).
- Two graduated glass micropipettes (0.1 mL capacity; 0.001 mL precision) were used, positioned horizontally. These were connected to the specimen by means of silicone tubing to measure the amount of fluid passing through the implant–abutment interface.

#### Evaluation of Microleakage

1. *System tightness.* Adequate system tightness was essential to perform the perfusion test. A negative control test was performed to verify system integrity. Two non-perforated implant dummies were sealed into the block with cyanoacrylate adhesive. The system was pressurized for 24 h to ensure no leaks occurred at the implant-base interface or tubing connections. Once the tightness of the system was verified, the dummies were removed and the two implants were inserted following the previously described protocol.
2. *Perfusion procedure.* Filtration fluid was introduced to the system of implants without the abutments connected to eliminate trapped air. Once fluid continuity was established, the prosthetic abutments were connected. The fluid reservoir was positioned 34 cm above the specimen, generating a constant hydrostatic pressure of 25 mm Hg (34 cm H_2_O). An air bubble was introduced into each pipette using a dual-syringe method to serve as a displacement marker. Using one syringe, 0.25 cm^3^ of air was introduced into the tubing, while a second syringe aspirated filtration fluid to position the air bubble to the 0.000 reference mark of the pipette. At that point, both syringes were removed. The system was stabilized for 10 min. The position of the bubble was recorded, and a second measurement was taken after 30 min to calculate the mean displacement of the bubble. If perfusion exceeded 30% of the mean reference value, the condition was considered hyperfiltration, and the implant was removed and resealed.
3. *Reference perfusion.* Reference perfusion was performed using original Straumann^®^ abutments tightened to 35 N·cm. After a 10 min stabilization period of the air bubble at the 0.000 mark, the bubble position was recorded. A second measurement was taken after 24 h, corresponding to the baseline perfusion. Any deviation from this baseline during subsequent testing was attributed to microleakage at the implant–abutment interface.
4. *Experimental perfusion.* Two experimental tests were performed:
  - Test 1: Abutments tightened to 5 N·cm.
  - Test 2: Abutments tightened to the manufacturer-recommended torque of 35 N·cm.

Following the perfusion test in Test 2, the removal torque values were recorded to calculate torque loss.

### 2.6. Statistical Analysis

Statistical analyses were performed using IBM SPSS Statistics for Windows, Version 27.0 (IBM Corp., Armonk, NY, USA).Although measurements were performed on the same implants, each measurement was considered independently and repeated twice. Given the exploratory nature of this pilot study and the limited sample size, all measurements were treated as independent observations to allow for statistical analysis beyond descriptive evaluation.

Descriptive statistics were calculated for all study variables, all of which were continuous. Given the limited sample size and non-normal distribution, a nonparametric analytical approach was adopted. Differences in microleakage values between original and non-original abutments, as well as between multi-unit and screw-retained cementable abutments, were evaluated using the Mann–Whitney U test. Comparisons between microleakage values obtained at different torque levels (5 N·cm and 35 N·cm) were performed using the Wilcoxon test. The amount of microleakage and the percentage of torque loss was analyzed using the Spearman correlation coefficient. The coefficient of variation was calculated to assess the reproducibility of microleakage measurements. The level of statistical significance was set at 5% (α = 0.05).

A post hoc power analysis revealed large effect sizes (Cohen’s d ranging from 1.73 to 3.06), depending on the abutment type and torque condition, corresponding to statistical power values between 76.6% and 99.5%.

## 3. Results

### 3.1. Data Acquisition and Exclusions

A total of 74 microleakage measurements were recorded. Deviations from the protocol occurred in two instances: the non-original A single Ibodontit multi-unit abutment fractured during the tightening phase (Table 2), at the upper portion of the fixation screw. As measurements were performed in duplicate, this event precluded the completion of two measurements on Implant 1. Additionally, Implant 2 exhibited aberrant, markedly elevated microleakage values during the final phase of the study compared with baseline measurements. Consequently, these data points were deemed invalid, and the implant was excluded from the final analysis. This explains the absence of measurements in Table 3. Due to the study timeline, two measurements corresponding to screw-retained cementable abutments (Ticare and NP Prótesis S.L.) could not be repeated (Table 3).

### 3.2. Microleakage Analysis by Abutment Type and Torque

**Figure 2:**
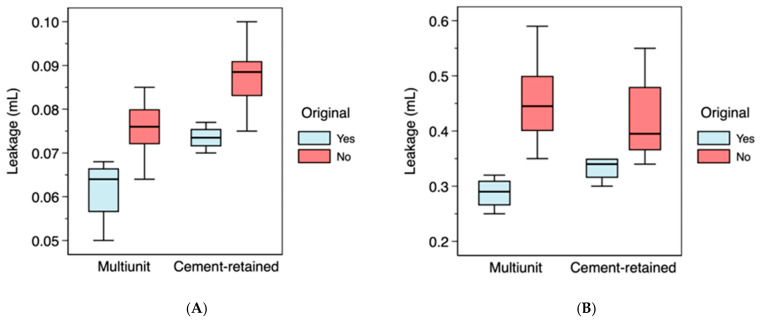
(**A**) Distribution of microleakage measurements for the different abutment types with a torque of 35 N·cm. (**B**) Distribution of microleakage measurements for the different abutment types with a torque of 5 N·cm.

Microleakage was detected in all abutment groups. Under all conditions, the lowest microleakage values were recorded for original Straumann^®^ abutments tightened to the recommended torque of 35 N·cm. At 24 h, mean microleakage values were 0.062 ± 0.008 mL for multi-unit abutments and 0.074 ± 0.003 mL for screw-retained cementable abutments. Conversely, non-original abutments exhibited significantly higher microleakage values, with mean values of 0.076 ± 0.006 mL for multi-unit abutments and 0.087 ± 0.007 mL for screw-retained cementable abutments (Figure 2A).

Reducing the installation torque to 5 N·cm resulted in a significant increase in microleakage across all groups compared with the manufacturer-recommended tightening torque of 35 N·cm. At 5 N·cm, original abutments showed mean microleakage values of 0.288 ± 0.030 mL for multi-unit abutments, and 0.333 ± 0.024 mL for screw-retained cementable abutments. Non-original abutments at 5 N·cm also demonstrated elevated values of 0.455 ± 0.071 mL for multi-unit abutments, and 0.421 ± 0.068 mL for screw-retained cementable abutments (Figure 2B).

Statistical analysis revealed that at both torque levels (5 N·cm and 35 N·cm), microleakage was significantly greater in non-original abutments than in original abutments (*p* < 0.01), regardless of the prosthetic connection type.

### 3.3. Comparison of Microleakage Between Multi-Unit and Screw-Retained Cementable Abutments

At the suboptimal torque of 5 N·cm, original screw-retained cementable abutments exhibited greater microleakage than multi-unit abutments, though this difference did not reach statistical significance (*p* = 0.057). Among non-original abutments at 5 N·cm, no statistically significant differences were observed between prosthetic connection type (*p* = 0.174). However, at 35 N·cm, statistically significant differences were found between multi-unit and screw-retained cementable abutments for both original (*p* = 0.029) and non-original abutments (*p* < 0.01).

### 3.4. Influence of Torque on Microleakage

**Figure 3:**
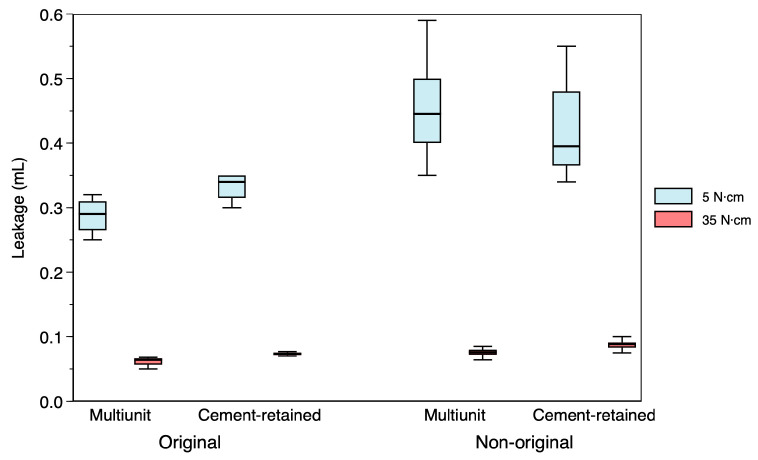
Distribution of microleakage measurements at 5 N·cm and 35 N·cm.

When comparing microleakage values obtained at different torque values (5 N·cm versus 35 N·cm), statistically significant differences were found in all conditions (*p* < 0.01), indicating that microleakage was significantly lower when the manufacturer-recommended tightening torque was applied (Figure 3).

### 3.5. Torque Loss Analysis

**Figure 4:**
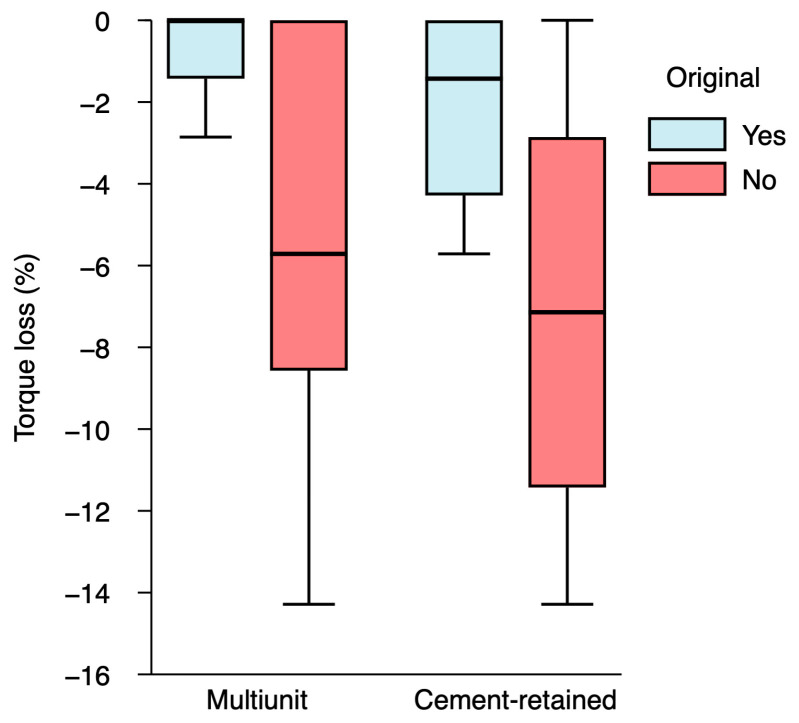
Distribution of torque loss among the different abutment types.

Analysis of removal torque revealed that 57.1% of multi-unit abutments experienced torque loss, with a median reduction of −2.86% (interquartile range [IQR]: −1.43 to 0.00), considering the applied torque of 35 N·cm as 100%. Torque loss was more prevalent and severe in screw-retained cementable abutments, affecting 77.8% of samples with a median reduction of −5.71% (IQR: −8.57 to −2.86). Non-original abutments exhibited significantly greater torque loss compared with original abutments (*p* < 0.01). No statistically significant differences in torque loss were observed between multi-unit and screw-retained cementable abutments (Figure 4).

### 3.6. Correlation Between Microleakage and Torque Loss

**Figure 5:**
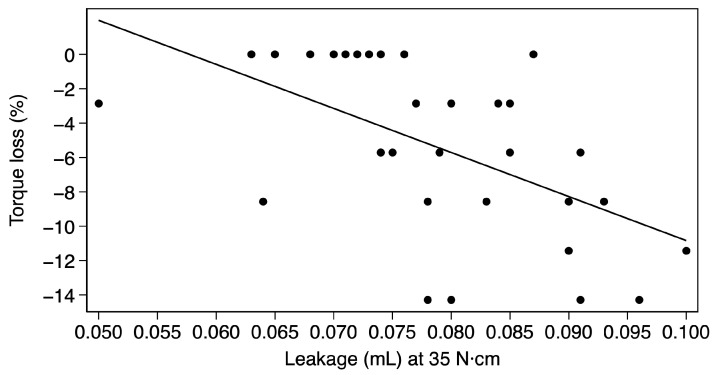
Distribution of microleakage as a function of torque loss.

Given the absence of statistically significant differences in torque loss between multi-unit and cementable abutments, a combined analysis was performed to evaluate the correlation between microleakage and torque loss. A statistically significant linear correlation was observed between microleakage and torque loss at 35 N·cm (R = −0.614). This relationship suggests that torque loss increases by approximately 2.56 percentage points for each additional 0.01 mL of microleakage (Figure 5).

### 3.7. Reproducibility of the Perfusion-Based Method

Finally, as this perfusion-based method represents a novel approach for quantifying microleakage at the implant–abutment interface, its reproducibility was assessed. This was done by performing 10 repeated measurements on an original Straumann^®^ multi-unit abutment tightened to 35 N·cm. Reproducibility analysis (n = 10) showed a mean microleakage value of 0.0625 mL (SD = 0.0071), with a median of 0.0630 mL (IQR: 0.0580–0.0670) and a range from 0.0500 to 0.0750 mL, corresponding to a coefficient of variation of 11.4%.

## 4. Discussion

Current available studies identify the penetration of oral microorganisms into the implant–abutment interface as a significant risk factor for peri-implant tissue inflammation and marginal bone loss, thereby compromising the long-term success of implant-supported restorations [6,7,8,11,18]. In the present in vitro study, microleakage and abutment screw torque loss were evaluated at varying tightening torques in original abutments and their corresponding non-original (compatible) counterparts within a mixed internal connection implant system.

Non-original abutments exhibited significantly greater microleakage than original abutments at both tested torque levels (5 N·cm and 35 N·cm), regardless of the prosthetic connection type (multi-unit or screw-retained cementable). These findings are consistent with those reported by Berberi et al., who utilized rhodamine dye to quantify leakage in Astra Tech implants [27]. They similarly observed that non-original abutments demonstrated superior leakage values compared with original components. This disparity is likely attributable to differences in marginal precision and manufacturing tolerances [33]. Non-original components may present morphological deviations from the proprietary design, resulting in an increased microgap and, consequently, greater fluid transport. This is supported by Mattheos et al. [34], who identified marked quantitative and qualitative discrepancies between original and non-original abutments connected to Straumann TL implants.

The mechanical integrity of the implant–abutment interface relies heavily on friction between matching surfaces to resist micromovements under functional loading. Decreased friction correlates with decreased preload on the retention screw [35], potentially exacerbating micromovement. Rismanchian et al. compared *E. coli* microleakage in Straumann^®^ SP implants with original Solid, SynOcta, and Cast On (Straumann^®^) abutments and non-original CastableRhein (Rhein 83) abutments [36]. Despite showing significant differences with better fit in the original abutments, they found no differences in the number of filtered colonies (CFU/mL), concluding that the relationship between the amount of microleakage and the size of the microgap was not statistically significant. In contrast, Solá-Ruiz et al. utilized scanning electron microscopy (SEM) to evaluate the vertical discrepancy of the IAI across five implant brands (Biofit, Bioner S.A., 3i Biomet, BTI^®^, and Nobel Biocare^®^) with external hexagonal connections, concluding that, while microgaps exist, many fall within a clinically acceptable range (3.46 ± 2.96 µm) [37]. However, the present study suggests that in terms of fluid dynamics, even small discrepancies in non-original components result in significantly higher volume transport.

Torque application proved to be a critical determinant of seal integrity. Microleakage volume increased significantly in all groups when the torque was suboptimal (5 N·cm). This aligns with the consensus in the literature that torque magnitude dictates the coupling intimacy and permeability of the connection. Larrucea et al. [19] studied the fit and microleakage of the IAI at <10 N·cm, 10 N·cm, 20 N·cm, and 30 N·cm. They demonstrated that torque values <10 N·cm and 10 N·cm resulted in a lack of fit detectable by micro-CT and were associated with microleakage of *Porphyromonas gingivalis*. Furthermore, the architecture of the abutment plays a role; screw-retained cementable abutments exhibited significantly higher microleakage than multi-unit abutments. This may be due to the internal fixation screw creating an additional interface for potential leakage.

The most frequent mechanical complication in single-unit implants is loosening of the fixation screw. This results from different factors such as a reduced preload at the connection, torque variations, screw characteristics [38], type of connections [39], inadequate fit at the IAI [12,13], and functional loads supported by the system [40]. When evaluating torque loss in abutments placed according to the manufacturer’s recommendations (35 N·cm), 57.1% of the multi-unit abutments reduced their initial torque by 2.86% (IQR: −1.43 to 0.00). In screw-retained cementable abutments, this occurred with greater frequency and severity, reaching 77.8% and 5.71% (IQR: −8.57 to −2.86), respectively. Likewise, non-original abutments exhibited significantly greater torque loss values (*p* < 0.01), with no differences between multi-unit and screw-retained cementable abutments.

In this study, the correlation between microleakage and torque loss was linear and moderately strong (R = −0.614), suggesting that for every 0.01 mL increase in microleakage, torque loss increases by approximately 2.56%. This finding aligns with Sahin et al., the only other study, to date, utilizing a modified perfusion method to correlate these variables [15]. Unlike the present study, Sahin et al. performed perfusion by connecting the system directly to the abutment. They examined two internal hexagonal connections, one titanium and one zirconia, and one internal conical titanium connection with abutments tightened to 25 N·cm. The greatest microleakage and screw torque loss were detected in the zirconia internal hexagonal connection, whereas no statistically significant differences were observed in the other two connections.

The phenomenon of “settling” is central to interpreting these results. As described by Kim et al. and Dailey et al., tightening torque causes the flattening of surface microroughness between the implant and abutment [38,39]. When this settling exceeds the screw’s elastic elongation, clamping force is lost. The high torque loss observed in non-original abutments suggests that their surface finish or material hardness may promote excessive settling or friction loss. This wear mechanism likely contributed to the fracture of the Ibodontit abutment and the anomalous data from Implant 2; repeated tightening cycles wear down the interface, altering friction coefficients and fit [40,41].

Several limitations must be acknowledged. Microleakage measurement was carried out on a small sample, considering all measurements as independent samples in order to perform analyses beyond the descriptive level. This approach allowed evaluation of the perfusion-based system under controlled conditions as an initial step before incorporating additional techniques that will enable a more detailed analysis of the implant–abutment interface. As noted, repeated loosening and tightening can alter surface topography, potentially influencing subsequent microleakage and torque loss measurements. Crucially, while the hydraulic conductance method offers a quantitative approach to microleakage, the reproducibility of the method was low (Coefficient of Variation: 11.4%). This variability may be attributed to the difficulty in eliminating all trapped air bubbles within the complex internal threads of the implant system or subtle variations in the hydrostatic pressure seal. This suggests that while the method is sensitive, strictly controlled protocols are required to minimize experimental error, and results should be interpreted with caution. The apical perforation of the implants, while necessary for the hydraulic conductance model, may have introduced micro-structural changes. Furthermore, the study was conducted under static conditions and does not reproduce the complexity of the clinical environment. In this regard, future studies should incorporate cyclic loading of the implant–abutment assembly, together with bacterial microleakage assessment and combined evaluation using hydraulic conductance and micro-computed tomography. This stepwise approach would allow a more comprehensive evaluation of the implant–abutment interface under conditions that more closely resemble the clinical scenario.

## 5. Conclusions

Within the limitations of this in vitro study, the following conclusions were drawn:

- Original abutments demonstrated significantly superior sealing capacity compared with non-original (compatible) abutments, regardless of the torque applied.
- Under all conditions—across both original and non-original, multi-unit and cementable abutment designs—microleakage was significantly minimized at the manufacturer-recommended torque (35 N·cm) compared with suboptimal torque (5 N·cm).
- A significant linear correlation was observed between microleakage volume and torque loss. While the specific abutment design (multi-unit vs. screw-retained) did not significantly influence torque maintenance, abutment originality was the decisive factor, with non-original components exhibiting significantly greater torque loss.
- While microleakage values were comparable in multi-unit and cementable abutments at 5 N·cm, design differences became apparent at the recommended torque. At 35 N·cm, screw-retained cementable abutments exhibited significantly higher microleakage than multi-unit abutments, even within the original group.
- The hydraulic conductance perfusion system proved to be a sensitive tool for the quantitative assessment of fluid transport at the implant–abutment interface, identifying distinct differences between component types.

Consequently, and taking the results into account, it can be concluded that, for the system evaluated, the use of original abutments ensures superior sealing and mechanical stability at the connection. Consequently, original components may offer better long-term clinical outcomes by mitigating the risks of screw loosening and biologic complications associated with microleakage, compared with non-original alternatives.

## Figures and Tables

**Table 1:** Specifications of multi-unit and screw-retained cementable abutments.

Prosthetic Abutment	Manufacturing	Origin	Brand	Gingival Height	Instruments Used
Multi-unit abutment	Standard	Original	Straumann^®^	2.5 mm	Ratchet, torque wrench, and screwdriver (SCS Straumann^®^)
	CAD/CAM	Non-original	Medentika^®^ (Straumann Group)	3.5 mm	Ratchet with torque wrench (Thommen Medical^®^) and multi-unit key (Nobel Biocare^®^)
	CAD/CAM	Non-original	Smart Implant Solutions S.L.	2.5 mm	Ratchet and torque wrench
	CAD/CAM	Non-original	Ibodontit S.L.	2.5 mm	Ratchet and torque wrench
Screw-retained cementable abutment	Standard	Original	Straumann^®^	3 mm	Original screw, ratchet, torque wrench, and screwdriver (SCS Straumann^®^)
	CAD/CAM	Non-original	Createch Medical S.L. (Straumann Group)		Compatible screws, ratchet, torque wrench, and screwdriver (SCS Straumann^®^)
	CAD/CAM	Non-original	New Ancorvis S.L. (Thommen Medical^®^)		Compatible screws, ratchet and torque wrench
	CAD/CAM	Non-original	Mozo-Grau S.A.		Compatible screws, ratchet and torque wrench
	CAD/CAM	Non-original	NP Prótesis S.L.		Compatible screws, ratchet and torque wrench

**Table 2:** Microleakage in multi-unit abutments.

Prosthetic Abutment	Implant 1 (5 N·cm)	Implant 1 (35 N·cm)	Implant 2 (5 N·cm)	Implant 2 (35 N·cm)
Straumann^®^ (original)	0.32	0.063	0.25	0.068
	0.28	0.050	0.30	0.065
Medentika^®^ (compatible)	0.39	0.078	0.35	0.074
	0.40	0.072	0.41	0.064
Smart Implant Solutions (non-original)	0.50	0.074	0.40	0.080
	0.46	0.078	0.43	0.071
Ibodontit (non-original)	0.54	X	0.49	0.080
	0.50	X	0.59	0.085

X: Abutment fracture.

**Table 3:** Microleakage in screw-retained cementable abutments.

Prosthetic Abutment	Implant 1 (5 N·cm)	Implant 1 (35 N·cm)	Implant 2 (5 N·cm)	Implant 2 (35 N·cm)
Straumann^®^ (original)	0.33	0.070	0.35	0.077
	0.35	0.074	0.30	0.073
Createch Medical (non-original)	0.34	0.087	0.42	0.091
	0.38	0.090	0.36	0.085
Ticare (non-original)	0.35	0.076	0.38	0.079
	0.37	0.075	0.35	—
New Ancorvis (non-original)	0.38	0.084	0.48	0.090
	0.41	0.091	0.46	0.083

—: Measurement could not be performed.

## Data Availability

The original contributions presented in this study are included in the article. Further inquiries can be directed to the corresponding author.

## Abbreviations

The following abbreviations are used in this manuscript:

CAD/CAM	Computer-Aided Design/Computer-Aided Manufacturing
CFUs	Colony Forming Units
HSS	High-Speed Steel
IAI	Implant–Abutment Interface
IQR	Interquartile Range
SEM	Scanning Electron Microscopy
SPSS	Statistical Package for the Social Sciences
